# Supplementary material for: Are carbon emissions trading and green financial instruments synergistic? -Comprehensive quantitative research based on content analysis
Source: PLoS One. 2024 Mar 7;19(3):e0298601. doi: 10.1371/journal.pone.0298601 (PMC10919601; doi:10.1371/journal.pone.0298601)
Supplement: S1 Data — (ZIP) [file pone.0298601.s001.zip › Data/地级市Z_F工作报告.zip!/╔╜╢1⁄2/╝├─╧╩╨/└┤╫╘╣1⁄2╓┌║┼í╛┬φ┐╦╩2╛▌═°í┐_2007.docx]

各位代表：

　　现在，我代表市人民政府向大会报告工作，请予审议，并请各位政协委员和其他列席人员提出意见。

　　一、2006年工作回顾

　　2006年，是我市全面实施“十一五”规划、扎实推进省会现代化建设取得显着成效的一年。全市人民以邓小平理论和“三个代表”重要思想为指导，全面落实科学发展观，着力构建社会主义和谐社会，坚决贯彻国家宏观调控政策，深入实践“发挥省城优势，发展省会经济”总体思路，求真务实，干事创业，推动经济社会发展朝着又好又快的方向迈出坚实步伐。初步核算，实现生产总值2185.1亿元，增长15.7%；地方财政一般预算收入128.4亿元，增长21%。完成了市十三届人大四次会议确定的目标任务。

　　(一)新农村建设扎实推进。坚持把“三农”问题放在经济社会发展全局突出位置，实施一系列支农惠农措施，推动新农村建设迈出坚实步伐。农民人均纯收入5480元，增长13.9%，连续三年保持两位数增长，并高于城市居民收入增幅。转移农村劳动力18万人次，9.8万农村贫困人口实现脱贫。实现城乡用电同网同价，农电价格下降22.9%。市财政“三农”投入8.6亿元，增长41.5%。粮食直补、良种补贴、农机具购置补贴、农资增支综合直补全部落实到位。支农投入、农民增收“1020”和“6521”工程提前一年完成。粮食总产267.9万吨，连续四年获得丰收。蔬菜、畜牧和林果业比重分别达32.4%、34.3%和9.3%。农田水利设施继续完善，防灾减灾能力明显增强。大力推进“路水电气”等农村设施建设，村村通油路(水泥路)比率、自来水普及率、村内道路硬化率、卫生厕所普及率分别达96.8%、76.5%、71%和69%，“一建三改”农户增长37.7%。新型农村合作医疗参合率、农村低保覆盖率达91%和85.7%，农村教育、医疗、社会保障加快改善。大力推动近郊、远郊基础设施建设和产业发展，加大对财政困难县转移支付力度，县域经济实力有新的增强。

　　(二)经济运行质量继续提高。坚持以结构调整促进总量平衡和效益提升。三次产业比例调整为6.6∶45.9∶47.5。工业增加值增长19.3%，六大产业集群支撑作用继续增强。服务业增加值增长15.6%，现代服务业占服务业比重提高2.5个百分点。“三个亮点”继续加强，非公经济比重、高新产业比重、经济外向度分别达40.2%、32.3%和16%。在1016.8亿元固定资产投资中，投向农业、服务业和社会事业分别为32.9亿元、600.7亿元和137.5亿元，分别增长32.2%、29.1%和28%。社会消费品零售总额939.3亿元，增长16.3%。地域财政占生产总值比重、税收占地方财政比重都有新的提高。规模以上工业经济效益综合指数为238.8%，利税、利润分别增长24.9%、27.7%。金融机构各项存贷余额分别为4024.6亿元和3788.4亿元，增长17.7%和18.3%。煤电油运支撑条件继续改善，经济薄弱环节得到加强。结构调整继续朝着国家宏观调控的方向扎实推进。

　　(三)经济增长方式加快转变。以自主创新为动力，以节能降耗减排为抓手，资源节约型、环境友好型社会建设迈出新步伐。实施创新型城市建设规划纲要及配套政策，构建起技术创新、创业服务、成果转化、资源共享、创新载体“五大平台”，启动组织、评价、规划、责任、政策、主体“六大体系”，研发经费占生产总值比重、授权专利增长率分别达1.9%和45.7%，省级以上企业技术中心、工程技术研究中心分别为42个和29个，增长16.7%和141.7%。实施农田开发复垦整理，加强了节约用地管理。节能环保评价体系全面建立，循环经济扎实推进，百户重点用能企业节能降耗取得新成效。万元生产总值综合能耗、二氧化硫和化学需氧量排放量明显降低。创建环保模范城取得重大成效，环保投资指数2.32%，提高0.21个百分点，市区部分河段截污治污工程基本完成，加强扬尘、机动车尾气治理，空气良好天数比率84.1%，创有监测以来最好纪录。

　　(四)社会事业加快发展。推进公共财政向社会事业倾斜，加快基本公共服务均等化步伐。市财政社会事业支出20.3亿元，增长20.8%。“两免一补”全面落实，建立起教师工资、公用经费保障及校舍维修改造长效机制，校舍标准化建设稳步推进。教育布局继续优化，基础教育、职业教育、成人教育协调发展，教育乱收费问题得到有效治理。高标准完成34所乡镇卫生院改造，设立社区卫生服务机构126所，城乡卫生服务体系不断完善。创作了儿童剧《宝贝儿》等一批优秀文艺作品，各类文化遗产保护取得重要进展。全民健身蓬勃开展，竞技体育不断发展，全运会筹备工作进展顺利。整体性人才资源开发迈出新步伐，人才结构明显优化。人口自然增长率3.11‰，继续保持低生育低增长势头。人工增雨气象服务成效显着。涌现阳光大姐等一批便民服务品牌。残疾人事业、慈善事业全面发展。新闻出版、广播电视、社会科学、民族宗教、档案史志、妇女儿童、老龄、地震等工作均取得新的成绩。

　　(五)城市功能加快提升。全面加强城市规划建设管理，推动城市建设更好地支撑发展、服务市民、带动农村。城市总体规划上报国务院待批，土地规划修编全面启动，中心城区50多个片区控规修编完成70%，开展了新农村建设规划试点，“南控”和“北跨”规划编研取得阶段性成果，规划引导调控作用明显增强。城建投资380亿元，增长46%。奥体中心、鹊山龙湖、北园大街等重点工程全面展开，大明湖、泉城广场周边综合整治加快推进，完成市区45条主次干道整修改造，“四路一桥”建成通车，城市建设取得较大进展。“两级政府、三级管理、四级网络”管理体系日趋完善，管理重心下移和行政综合执法迈出新步伐。多措并举加大名泉保护力度，趵突泉实现三年连续喷涌。加强城市应急管理，提升快速反应能力，城市公共服务效率明显提高，成为全国优先发展公交示范城市。完善旧城改造运行机制，加强城乡环境综合整治，完成市区2499条背街小巷和55个居民小区整治任务。省会城市群经济圈规划建设扎实推进。人民群众对城市规划建设管理满意度有新的提高。

　　(六)改革开放水平逐步提高。坚持以深化改革、扩大开放推动科学发展，发展活力进一步增强。财税体制改革深入实施，促进财政收入实现质的提升。政府资金管理制度改革成效显着，累计11万笔收支项目未发生违规违纪问题，节支8600万元。投融资体制日趋完善，支撑发展和化解风险能力明显增强。行政事业资产管理制度改革取得阶段成效，行政国有资产得到有效整合。通过行政管理体制改革，促进行政行为规范、行政成本降低，初步构筑起源头防治腐败机制。首批列入监管的255户企业完成改制 93.3%，轻骑、济啤、济锅等重点企业实现资产重组，9家上市公司完成股权分置改革，国企改革推进力度、规范程度、综合成效全面提高。农村综合改革全面展开，乡镇机构改革基本完成。供热、供水、污水处理行业股权改革和价格管理体制改革取得新进展。对外贸易迈出新步伐，进出口总值43.9亿美元，增长16.7%，出口24.4亿美元，增长37.2%。出口结构进一步优化，高新技术与机电产品出口比重提高8个百分点。引进市外资金561.4亿元，实际利用外资和外商直接投资增长24.2%和15.1%。“走出去”战略成效显着，对外工程承包、劳务合作合同额增长2.5倍。煤炭会、童博会、信博会、科技展交会等95个展会成功举办。服务业开放领域逐步扩大。外事、侨务、对台和友城工作取得新成绩。

　　(七)着力解决民生问题。城市居民人均可支配收入15340元，增长13%，居民消费价格指数100.9%，物价保持基本稳定，汽车、住房、文化、教育、旅游等支出比重提高。城镇就业再就业13.27万人次，城镇登记失业率3.87%。市财政投入社会保障9.2亿元，增长23.3%。企业社保基金收入38.2亿元，增长19%，基本养老、医疗、失业、工伤、生育保险参保人数分别增加5.9万、16.5万、1.7万、8万和1万。社会救助投入1.47亿元，增长58.8%，城乡低保标准普遍提高。城市居民人均住房使用面积20.1平方米，农村居民人均生活用房面积35.3平方米，分别增加0.3和1.5平方米，住房公积金和廉租住房制度加快推进，供给结构加快调整，住房价格基本平稳。森林覆盖率、建成区绿化覆盖率、污水处理率、集中供热普及率、垃圾无害化处理率均有新的提高，生活环境日益改善。

　　(八)社会环境更加和谐。坚持社会公平正义，注重夯实社会和谐基础，“和谐济南”建设迈出坚实步伐。深入贯彻《依法行政实施纲要》，在全国率先推出复议为民公开事项，全面推行行政执法责任制。“五五”普法全面启动，司法救助和法律援助扎实开展。政务公开逐步深化，电子政务加快推进，办理人大代表建议311件、政协提案603件，政府决策的社会参与程度不断提高。“平安济南”建设扎实推进，社会治安综合治理继续加强，维护稳定、打击犯罪、化解矛盾、治安防控能力全面提升，交通秩序继续改善，平安社区建设成效明显，公众安全感进一步提高。食品药品等各类市场秩序进一步规范，安全生产形势保持平稳，处置公共卫生事件能力不断增强。深入开展“弘扬济南精神、创建文明城市”主题行动，创城“百件实事”全面完成。创新行政服务手段，完善便民利民措施，发展环境逐步优化。强化源头治理腐败，监察审计全面加强，治理商业贿赂、查处腐败案件、纠正行业不正之风取得新进展。信访工作继续加强。国防教育、人民防空、征兵工作和双拥共建扎实推进，军政军民团结更加巩固。

　　各位代表，过去一年的发展和进步，是省委、省政府和市委正确领导的结果，是全市人民开拓进取、努力奋斗的结果，是人大、政协、各民主党派监督支持、团结协作的结果，是驻济单位、各界人士积极参与的结果。在此，我代表市政府向辛勤工作在各行各业的全体市民，向给予政府工作大力支持的各级人大代表、政协委员、各民主党派、工商联、各界人士，向关心支持省城工作的中央、省驻济单位，向关心帮助地方工作的驻济人民解放军、武警官兵，向为济南发展做出贡献的港澳台同胞、海外侨胞、国内外朋友们，表示崇高敬意和衷心感谢！

回顾近年工作，我们在推进科学发展的实践中，初步形成以下共识，这就是：始终坚持以科学发展观统领全局，紧紧抓住发展第一要务，坚持好中求快，把中央和省委、省政府重大部署与济南实际紧密结合，努力探索科学发展推进机制，加快增长方式转变，切实把经济发展建立在速度与结构、质量、效益、后劲相协调的基础上；始终坚持把构建和谐社会作为发展总目标，坚持以人为本，积极解决人民群众最关心、最直接、最现实的利益问题，注重社会公平和公共资源均衡配置，全面增强发展协调性，让全体人民共享改革发展成果；始终坚持从体制、机制和根本上解决发展中的问题，集中突破制约科学发展的突出矛盾，不断调整生产关系，努力构建促进科学发展、和谐发展的体制保障；始终坚持提高政府执行力和公信力，推进政府职能转变，提高依法行政水平，完善科学民主决策机制，加强社会管理和公共服务，努力建设务实、高效、廉洁政府。这些认识和体会，我们仍需继续坚持和发扬。

　　站在全国全省大局，我们与人民群众期望和先进城市相比，综合实力仍然不强，发展后劲仍然不足，对外开放水平仍然不高。具体表现在：一是经济结构调整步伐亟待加快。工业经济支撑带动作用尚不突出，县域经济仍较薄弱，非公经济、外向经济、高新产业发展步伐不快，先进制造业、现代服务业等新的增长点仍需加大培育力度。二是增长方式转变任务还很艰巨。自主创新能力亟待增强，全面实现节能降耗减排目标压力很大，能源、资源和环境问题仍是制约科学发展的突出矛盾。三是推进协调发展力度尚需加强。城乡区域发展差别较大，社会事业发展相对滞后，新农村建设任重道远，农民持续增收基础尚不稳固，就业、看病、上学、住房等关系群众切身利益的突出问题尚未解决好。四是推进科学发展的体制环境亟待改善。市场配置资源的基础性作用远未充分发挥，政府职能亟需深入转变，公共服务能力还不适应构建和谐社会的需要。这些问题，我们一定要高度重视，务必保持清醒头脑，找准差距和症结，采取更加有效措施努力加以解决。

　　二、2007年经济社会发展的指导思想和主要目标

　　今年是巩固扩大宏观调控成果，深入落实科学发展观，扎实推进和谐社会建设的重要一年。政府工作的指导思想是：坚持以邓小平理论和“三个代表”重要思想为指导，全面贯彻党的十六大以来各项方针政策，以科学发展观为统领，加快构建社会主义和谐社会，深入实践“发挥省城优势，发展省会经济”总体思路，以筹办第十一届全运会为契机，把握“四个关系”，突出“四个注重”，落实“四个着力”，促进“四个协调”，努力推动全市经济社会又好又快发展。

　　——把握“四个关系”。即全面认识和准确把握好与快、保与压、局部与全局、当前与长远的关系。特别要在“好”上下功夫，谋划与推进发展更加注重科学全面，努力增强发展的稳定性、协调性和可持续性。

　　——突出“四个注重”。即注重落实上级精神与实际工作紧密结合，找准着力点和突破口；注重用改革、创新、市场的办法从根本上解决矛盾，着力加强薄弱环节；注重决策、执行、监督体系建设，切实把各项决策部署落到实处；注重调动、保护、发挥各方面积极性，形成推动科学发展的整体合力。

　　——落实“四个着力”。即着力调整经济结构和转变增长方式，着力加强资源节约和环境保护，着力推进改革开放和自主创新，着力促进社会发展和解决民生问题。

　　——促进“四个协调”。即努力实现速度、质量、效益相协调，消费、投资、出口相协调，人口、资源、环境相协调，经济发展与社会民生相协调。

　　今年经济社会发展的主要预期目标：生产总值增长14%，其中三次产业分别增长5%、14%和15%，地方财政一般预算收入增长16%，社会消费品零售总额增长15%；全社会固定资产投资增长20%，其中社会事业投资增长25%；出口增长18%，外商直接投资增长20%；城市居民人均可支配收入、农民人均纯收入均增长10%，居民消费价格总水平涨幅控制在3%以内，城镇登记失业率控制在4.1%以内。主要约束性指标：万元生产总值综合能耗降低4.5%，二氧化硫、化学需氧量排放量降低1.6%和2.98%，环境空气质量良好率达85%，人口自然增长率控制在5.4‰以内。这些突破发展瓶颈的重要指标，必须竭尽全力加以完成。

　　各位代表，全面完成今年的任务目标，推动经济社会又好又快发展，机遇与挑战并存，综合条件总体有利：从发展趋势看，国家保持宏观政策的连续性和稳定性，对我市保持速度、优化结构、转变方式、提高效益都会产生积极影响。从发展基础看，经过几年积极探索和努力，全市推进科学发展的驾驭能力不断提高，体制政策不断完善，承载能力显着提升。从发展环境看，省委、省政府高度重视省会发展，社会各界对济南发展寄予厚望，广大干部群众建设美好家园的激情空前高涨。站在新的起点，面对新的机遇，我们有信心、有能力克服前进中的各种困难，圆满完成本届政府肩负的使命，推动省会现代化建设迈出新步伐，向全市人民和所有关心济南发展的各界人士交出合格答卷。

　　三、2007年经济社会发展的主要任务

　　围绕实现今年经济社会发展目标，重点做好八方面工作：

　　(一)着力壮大县域经济，扎实推进新农村建设。一是加大支农投入。财政预算内支农投入增长27%，着力实施农民增收、城镇建设、道路畅通、饮水安全、生态富民、造林绿化、医疗惠民、教育振兴、弱势保障、市场拓展“十大行动”，显着改善农村生产生活条件，提高发展承载力。农民人均纯收入力争突破6000元。二是大力发展现代农业。立足抗灾夺丰收，确保粮食安全“三条底线”。做大做强做优蔬菜、畜牧、林果等产业。实施优势农产品区域化布局，提升农业标准化、良种化、机械化和信息化水平，大力培育农村市场体系，发展农民专业合作经济组织，积极培养新型农民。规模以上农业龙头企业突破200家，带动更多农户纳入产业化经营。三是深化农村综合改革。落实中央“三个高于”支农惠农政策，健全“三农”投入稳定增长机制。加快培育农村金融市场，争取更多社会资金投向农业和农村。深化农村行政管理体制和县乡财政管理体制改革，提高依法行政和为农服务水平，确保农村义务教育经费保障机制改革落到实处。四是着力推进县域经济发展。把县域经济发展摆在更加突出的位置，加快基础设施向县域延伸，推进产业合理布局，着力提升县域经济产业层次和整体实力。加强分类指导，完善科学合理的评价体系。加大对困难县区转移支付力度，改进加强对县区协调服务，激发县域自身发展活力。

　　(二)大力推进经济结构调整，提高产业素质和竞争力。一是优化工业结构。继续加大工业投入，发展先进制造业，运用先进技术改造提升传统产业，增强六大产业集群辐射带动力，壮大一批成长性中小企业。实现规模以上工业增加值增长15%，销售收入、利税均增长18%。优先发展电子信息、生物制药、新材料等高新产业，高新技术产业比重达到34.3%。推动国家信息通讯国际创新园、中国服务外包基地城市和国家软件出口创新基地建设，使之成为带动产业结构升级的重要引擎。提升园区管理水平，提高投资强度与能耗环保标准，精心打造产业集聚、技术创新与环保节能示范区。二是加快发展服务业。大力发展金融、物流、会展、中介等生产性服务业，积极发展信息、文化等知识型服务业，繁荣发展旅游、商贸餐饮、社区服务等生活性服务业，努力提升服务业产业层次，进一步发挥服务生产、方便生活、增加就业的重要作用。加强房地产市场监管，引导房地产业健康发展。三是提高建筑业发展水平。规范建筑市场管理，推广环保节能建材和技术，拉长产业链条，壮大产业规模，显着提升建筑业综合效益。四是优化所有制结构。落实各项促进政策，放宽市场准入，鼓励非公经济进入社会事业和公用领域、参与国有资产重组，健全中小企业贷款担保机制和企业信用体系，推动骨干民营企业做大做强。非公经济比重达到44%。五是着力优化投资结构。严控高消耗、高污染和产能过剩行业扩张，引导资金重点投向农业、先进制造业、高新技术、现代服务业和社会事业。

　　(三)高度重视自主创新，大力推动增长方式转变。一是加快自主创新步伐。继续加大科技投入，研发经费占生产总值比重达2%。支持重点领域科技创新，加强重大科技项目攻关，实施重大科技专项，着力解决支柱产业竞争力不强、核心技术受制于人、资源环境矛盾加重等突出问题。深入落实创新型城市规划纲要和配套政策，发挥企业主体作用，着力打造产品品牌，加强知识产权保护，促进创新成果向现实生产力转化，推进“济南制造”向“济南创造”转变。二是促进重点行业节能降耗减排。安排专项引导资金，在钢铁、水泥、化工、建筑等领域实施一批节能示范项目，推动节能降耗迈出更大步伐。健全环保准入制度，落实减排目标责任，加强大气和固体废弃物污染防治，强化城市中心区、生态功能区、饮用水源地以及小清河流域污染防控措施，依法惩处各类环境违法行为。抓住列入省循环经济试点城市机遇，推动循环经济加快发展。落实最严格的土地管理制度，健全建设用地调节机制，严控增量，盘活存量，提高集约用地水平，确保耕地总量动态平衡。三是完善调控手段。实施能源、资源、环境规费政策，完善土地、水等重要资源产品价格形成机制，健全资源合理开发和生态恢复经济补偿机制，落实资源综合利用和废旧物资回收利用优惠政策，引导各行各业节能降耗、保护环境。完善相关政策措施，及时疏导资源价费改革带来的公用价格矛盾。倡导健康、节约、环保生产和消费，推进资源节约和环境友好型社会建设。

　　(四)加大公共财政倾斜力度，推进社会事业全面发展。一是促进教育公平。坚持教育优先发展，巩固提高基础教育，鼓励和规范发展职业教育、成人教育、民办教育。确保教职工工资发放、公用经费落实、贫困学生救助、农村校舍改造“四个到位”，巩固“两免一补”成果，并向城市低保家庭延伸，设立高中阶段奖学金和助学金，解决贫困家庭和外来务工人员子女上学难问题。坚决制止教育乱收费。二是推进公共卫生“五个覆盖”。基本完成乡镇卫生院改造，加强村级卫生室标准化建设，推进乡村卫生服务一体化管理，医疗服务体系覆盖农村；提高农民参合率、补助标准和筹资水平，新农合制度覆盖全体农民；弥补空白，健全网络，完善功能，社区卫生服务覆盖城区；加强公共卫生监测和应急机制建设，公共卫生服务和“120”急救网络覆盖城乡；落实“三个纳入”政策，中医药服务覆盖社区乡镇。加强医药价格监管，启动大病统筹为主的城镇居民合作医疗制度。做好人口计划生育工作，稳定人口低生育水平，提高出生人口素质，积极应对人口老龄化问题。三是加快发展文化事业和文化产业。完善公共文化服务体系，加快建设一批群众性文化设施，广泛开展城乡公益文化活动。深化文化体制改革，大力发展文化产业，鼓励非公资本依法进入文化领域。加强各类文化遗产保护，强化文化市场监管。四是积极发展体育事业。扎实推进全运会筹备工作，加快各类场馆、配套设施和城乡体育设施建设，全面提高全民健身和竞技体育水平。五是加强人才队伍建设。重点做好急需人才的培养引进，完善人才资源市场配置和评价机制，提高人力资源对科学发展支撑力。

　　(五)以筹办全运会为契机，加强城市规划建设管理。一是继续完善城市规划体系。着力深化各功能分区、重点片区和专项规划，实现重点区域和中心城区2000多平方公里控规全覆盖。扎实搞好新农村建设规划编制。深化省会城市群经济圈规划研究。二是加强重点工程和片区建设。推进沿经十路、沿小清河综合治理与纵深开发。加快奥体中心、唐冶、孙村和大学科技园等重点片区建设。积极推动大明湖、泉城广场周边地区综合整治。三是加强城市基础设施建设。对市区35条主次干道实施改造，基本完成北园路道路改造。加快绕城大北环、黄河三桥、泺口隧道等实施进度，推进北跨进程和东、西区基础设施配套。搞好公交场站枢纽及各类市政设施建设。四是大力推进棚户区改造。强化政府责任，制定相关政策，大力推进旧城改造，力争用三年左右时间基本完成棚户区改造。五是提升城市管理水平。实施城市管理系统数字化工程，完善“重心下移”管理体制，构建运转高效、保障有力的长效机制。扎实推进城乡环境综合整治，重点搞好市容环境19项专项整治。六是推进生态城市建设。加大环保模范城创建工作力度，重点加强城市环境空气质量整治、市区河道水系治理和饮用水源地保护，力争达到国家32项新指标要求。提高城市绿化覆盖率，实施荒山造林、退耕还林、平原绿化和绿色通道工程，推动南部山区保护与开发进程。落实“增雨、补源、置采、控流、节水”十字方针，健全保泉长效机制。

　　(六)推进重点领域和关键环节改革，提高对外开放水平。一是深化行政管理体制改革。继续完善政府资金管理、行政审批、行政事业资产管理、机关事务管理、机关事业工资制度和政府投融资体制，巩固扩大改革成果，完善高效规范的制度体系。二是深化国有企业改革。基本完成首批监管企业改革改制，加快市直机关所属企业改革改制，继续推进市政公用企业改革进程。完善国有资本出资人代表派出机制和经营预算体系，健全公司法人治理结构，全面提高国资监管水平。三是推进事业单位改革。以经营开发服务类事业单位改企转制为重点，完善配套政策，推进改革进程。发展各类中介组织，推动行政机构承担的社会职能向中介组织转移。四是转变外贸增长方式。大力优化进出口商品结构，着力培育出口品牌，扩大高新技术、机电产品出口规模。鼓励进口重要原材料、先进适用技术和关键设备。五是提高招商引资水平。把招商引资作为扩大开放的重要抓手，摆在更加突出的位置，坚持扩大规模与优化结构并重，突出抓好投资规模大、技术水平高、产业带动力强的外资项目。坚持“市外即外”方针，积极引进国内项目。强化企业招商主体地位，打造招商引资平台，坚持导向激励政策，按照“三个一批”要求，精心搞好项目储备包装。更新观念，转变作风，着力改善投资环境，切实解决项目落地难问题。六是扩大对外投资合作。支持更多企业对外承揽工程、开展劳务合作和境外投资，加快“走出去”步伐，推动对外交流合作迈出新的步伐。

　　(七)高度重视改善民生，让发展成果惠及城乡居民。一是千方百计扩大就业。落实有利于就业的产业、财税、金融等政策，加强职业技能培训，鼓励自主创业，推动大中专毕业生、退役军人创业就业和农村劳动力转移。大力开发公益性就业岗位，加大对零就业家庭等就业困难群体帮扶力度。全市城镇新增就业13万人次、转移农村劳动力16万人次。二是构建和谐劳动关系。着力提高低收入群体收入水平。严格执行最低工资制度，加强企业工资分配调控指导，实施农民工工资支付保证金制度，严查恶意拖欠工资行为。加强劳动用工合同管理，切实维护劳动者特别是农民工合法权益。三是加快社会保障体系建设。扩大社保覆盖范围，加强社保基金监管，城镇基本养老、医疗、失业、工伤、生育保险参保人数分别达到92万、86万、67万、74万和57万。扎实解决困难企业职工医疗保险问题，逐步把进城务工人员、被征地农民及灵活就业者纳入社会保障范围。四是完善城乡社会救助体系。抓好防灾减灾救灾工作，提高农村和城市低保标准，妥善解决困难群众和优抚对象实际困难。完成乡镇敬老院建设，五保户集中供养率提高到70%。支持发展福利和慈善事业，开展“五大救助工程”，提高特困群体救助水平。五是切实解决难点问题。加强经济适用房建设管理，完善城镇廉租住房制度，切实解决低收入家庭住房困难。坚决纠正土地征用、房屋拆迁、企业改制破产、基层执法中损害群众利益的问题。六是创造安全消费环境。深入整顿市场秩序，严厉打击食品、药品、农资制售假劣行为，推行明码实价，加强价格监督检查，深入治理违规收费，加强消费维权工作。

　　(八)推进“和谐济南”建设，营造和谐稳定社会环境。一是推进民主法制进程。加强法治政府建设，推进地方、行业、社区和基层依法治理，深入搞好“五五”普法，加强司法救助和法律援助，全面提升依法治市水平。完善村民、居民自治和企事业民主管理制度，推进民主决策、民主管理、民主监督。二是推进文明城市创建。深入开展社会主义荣辱观教育，培育文明道德风尚，加强青少年思想道德建设，弘扬“诚信、创新、和谐”济南精神，抓好“迎接十七大，创建文明城”主题行动，全面落实以市民文明、交通畅通、环境保护、综合治理、和谐社区、优化服务、城市平安、窗口明亮、为民解忧、文化建设为主要内容的“百件实事”，扎实解决群众普遍关注的问题。健全新型社区管理体制，加强社区服务设施建设。三是推进“平安济南”建设。继续加强双拥共建，增进军政军民团结，争创“双拥模范城”六连冠。落实维护社会稳定责任制，强化社会治安综合治理，加强政法基层建设投入，深入开展基层平安创建活动。毫不放松安全生产，强化防范措施，健全应急机制，重点加强矿山、危险品、消防、交通安全监管，严防重特大事故发生。四是正确处理和化解人民内部矛盾。高度重视人民来信来访工作，健全利益协调、诉求表达、矛盾调处、权益保障机制，落实领导干部接待和联系群众制度，畅通社情民意反映渠道，解决群众合理诉求，依法维护群众利益和社会稳定。

　　四、全面加强政府建设和管理创新

　　以建立公共服务型政府为方向，加快职能转变，推进管理创新，卓有成效地完成本届政府担负的任务。

　　(一)健全经济调节机制。完善经济调节职能，改进经济管理方式，确保国家宏观调控政策落到实处。切实把好规划、政策、土地、环评、能耗、安全等准入关口，加强投资方向引导，增强发展调控能力。理顺政府与市场、企业的关系，综合运用经济、法律以及必要的行政手段，完善社会信用体系和市场监管体系，健全引导激励约束机制，增强经济调节有效性。建立科学的绩效评价体系，把约束性指标纳入各级目标责任，加强督促检查和评价考核，切实把科学发展的要求落到实处。

　　(二)强化公共服务职能。改进行政管理方式，增强公共服务能力。改革和完善预算管理体制，优化财政支出结构，扩大公共产品和公共服务供给。进一步优化公共资源配置，逐步形成以基础教育、基本医疗、群众文化、公共设施为主要内容的公共服务体系。推动社会管理体制改革，支持社会组织和群众参与社会管理，形成政府主导、社会协同、公众参与的社会管理格局。健全应急预案体系，加强应急信息平台和队伍建设，加强人民防空工作，搞好人防设施建设，提高应对各种自然灾害和公共危机事件的能力。

　　(三)坚持科学民主决策。把科学民主决策作为行政基本制度，完善决策、执行、监督相协调的运行机制。健全重大决策集体研究和民主协商制度，自觉接受人大监督，充分发挥人民政协、民主党派及各界人士参政议政和民主监督作用。建立政策评价机制，凡发展改革重大决策，认真履行专家咨询、技术论证和决策评估程序，确保决策符合法律和民意。完善社会公示与听证制度，重点抓好涉及群众切身利益事项的听证，充分尊重群众知情权、参与权、表达权、监督权。健全行政决策反馈机制和责任追究制度，对决策失误造成重大损失的，追究相关领导和当事人责任。

　　(四)着力推进依法行政。制定依法行政各项制度，逐步将行政管理纳入依法运转轨道。提高政府规章和规范性文件质量，建立定期清理制度。健全行政执法主体资格制度，深化行政综合执法和相对集中行政处罚权改革，推行行政处罚自由裁量权基本标准制度，提高依法行政能力。落实复议为民公开事项，积极化解行政争议。严格落实行政执法错案追究、行政赔偿等项制度。严查执法不公、执法违法和行政不作为行为，切实做到有权必有责、用权受监督、违法要追究、侵权要赔偿。

　　(五)规范行政权力运行。高度重视廉政建设，着力构筑惩治和预防腐败体系，有效防止滥用公共权力。深化行政管理体制各项改革，完善堵塞滋生腐败漏洞的制度体系。继续深化政务公开，推进电子政务建设，提高决策透明度，拓宽公众监督渠道。充分发挥监察审计职能作用，健全发现和惩戒机制，支持其依法独立履行职责。全面治理商业贿赂，重点抓好工程建设、土地出让、产权交易、医药购销、政府采购、资源开发经销六个领域。对涉及公务员的商业贿赂，一查到底，依法惩处，决不手软。

　　(六)切实加大落实力度。全面深刻把握中央重大战略思想和省委、省政府重大部署，加强现代知识学习，提高驾驭市场经济、管理社会、依法办事能力。按照胡锦涛总书记的要求，全面加强八方面作风建设和思想道德修养，牢记“两个务必”，增强忧患意识、公仆意识和节俭意识，弘扬求真务实、艰苦奋斗作风。抓住影响科学发展和社会民生的主要矛盾，勇于正视困难，善于突破难点，务求取得实效。建立严格的目标责任制，坚持奖优、治庸、罚劣，鼓励干事创业，确保兑现向人民群众作出的承诺。

　　各位代表，加快建设实力强大、人民富裕、社会和谐、生态良好的现代化省会城市，是广大群众的共同愿望，是人民政府义不容辞的责任。我们要更加紧密地团结在以胡锦涛同志为总书记的党中央周围，高举邓小平理论和“三个代表”重要思想伟大旗帜，全面落实科学发展观、加快构建社会主义和谐社会，在省委、省政府和市委坚强领导下，把握新机遇，谱写新篇章，努力开创省会现代化建设的新局面！
